# Pharmacokinetics of tramadol after subcutaneous administration in a critically ill population and in a healthy cohort

**DOI:** 10.1186/1471-2253-14-33

**Published:** 2014-05-12

**Authors:** Neil M Dooney, Krishnaswamy Sundararajan, Tharapriya Ramkumar, Andrew A Somogyi, Richard N Upton, Jennifer Ong, Stephanie N O’Connor, Marianne J Chapman, Guy L Ludbrook

**Affiliations:** 1 Department of Anaesthesia, Pain Medicine and Hyperbaric Medicine, Royal Adelaide Hospital, Adelaide, SA 5000, Australia; 2 Intensive Care Unit, Royal Adelaide Hospital, Adelaide, Australia; 3 Discipline of Pharmacology, Faculty of Health Sciences, University of Adelaide, Adelaide, Australia; 4 Discipline of Acute Care Medicine, University of Adelaide, Adelaide, Australia; 5 Discipline of Pharmacometrics, Division of Health Sciences, University of South Australia, Adelaide, Australia

**Keywords:** Tramadol, Subcutaneous, Pharmacokinetics, Severely ill, Healthy subjects

## Abstract

**Background:**

Tramadol is an atypical centrally acting analgesic agent available as both oral and parenteral preparations. For patients who are unable to take tramadol orally, the subcutaneous route of administration offers an easy alternative to intravenous or intramuscular routes. This study aimed to characterise the absorption pharmacokinetics of a single subcutaneous dose of tramadol in severely ill patients and in healthy subjects.

**Methods/design:**

Blood samples (5 ml) taken at intervals from 2 minutes to 24 hours after a subcutaneous dose of tramadol (50 mg) in 15 patients (13 male, two female) and eight healthy male subjects were assayed using high performance liquid chromatography. Pharmacokinetic parameters were derived using a non-compartmental approach.

**Results:**

There were no statistically significant differences between the two groups in the following parameters (mean ± SD): maximum venous concentration 0.44 ± 0.18 (patients) vs. 0.47 ± 0.13 (healthy volunteers) mcg/ml (p = 0.67); area under the plasma concentration-time curve 177 ± 109 (patients) vs. 175 ± 75 (healthy volunteers) mcg/ml*min (p = 0.96); time to maximum venous concentration 23.3 ± 2 (patients) vs. 20.6 ± 18.8 (healthy volunteers) minutes (p = 0.73) and mean residence time 463 ± 233 (patients) vs. 466 ± 224 (healthy volunteers) minutes (p = 0.97).

**Conclusions:**

The similar time to maximum venous concentration and mean residence time suggest similar absorption rates between the two groups. These results indicate that the same dosing regimens for subcutaneous tramadol administration may therefore be used in both healthy subjects and severely ill patients.

**Trial registration:**

ACTRN12611001018909

## Background

Tramadol is an atypical centrally acting analgesic agent available as both oral and parenteral preparations. If given parenterally, the intravenous (IV) route is commonly preferred. However, administration of large IV bolus doses of tramadol can result in a high incidence of nausea and vomiting, which may be decreased by reducing the rate of delivery or by administering the drug via the subcutaneous (SC) route [1,2]. IV administration also requires venous access which is associated with infective and thromboembolic complications. Furthermore, while the intramuscular (IM) route is another alternative, SC delivery is preferred by patients [3,4]. A SC cannula can be sited for repeated needleless injections, reducing patient discomfort and increasing patient and staff safety [5]. Therefore, for patients who are unable to take tramadol orally, the SC route of administration offers an easy alternative. Although the pharmacokinetic profile of tramadol has been previously characterised in humans after oral, IM, IV administration, similar data are not available for subcutaneous tramadol and cannot be extrapolated from these studies [6-9].

While tramadol as the sole analgesic agent for the management of moderate to severe acute pain may be inadequate, when used as part of a multimodal analgesic regimen in severely ill and other patients it may offer several advantages [10]. Compared with pure opioid agonists at equianalgesic doses, tramadol is significantly less likely to lead to respiratory depression and has less effect on gastrointestinal motor function than morphine, whilst nausea and vomiting are the most common adverse effects and occur at rates similar to other opioids [1,11-17].

It is important to understand the pharmacokinetics of tramadol after SC injection, especially the rate of absorption and time to peak blood concentration, to determine the optimal dose and frequency of administration. It is also necessary to determine if there are differences between healthy subjects and severely ill patients in order to know if different dose regimens are needed in the healthy postoperative patients and in the unstable patients in the high dependency area.

The aim of this study was therefore to evaluate the pharmacokinetics of 50 mg tramadol administered as a single SC bolus dose to healthy opioid-naïve subjects and to severely ill patients in a high dependency setting. Evaluating the absorption kinetics for tramadol in healthy subjects and in patients will also assist in clinical decision-making and a better understanding of whether dosing regimens based on results from studies in healthy subjects may be transferrable to patients.

## Methods/design

Following approval from the Royal Adelaide Hospital Human Research Ethics Committee (approval numbers: patients - 90719; healthy subjects - 80717) and written informed consent, eight healthy male subjects and fifteen patients in the high dependency unit were enrolled in the study which was conducted separately for healthy subjects and patient in accordance with National Health and Medical Research Council of Australia guidelines.

### Healthy subjects

Healthy male subjects aged 18 to 65 years (inclusive) were recruited by advertisement in the university community. Body weight was greater than 50 kg and body mass index (BMI) was between 19 and 30 kg/m^2^. There was a requirement for adequate venous access in the left or right arm to allow collection of a number of blood samples. Health status was ascertained a week prior to the study by medical interview, physical examination and laboratory investigations including haematology, coagulation, biochemistry, liver function and urine drug screen (DipScan, Point of Care Diagnostics Australia Pty Ltd, Artarmon, NSW). All subjects were followed up one week after the study, when they repeated the medical interview and examination and reported any adverse events following the study.

### Patients

Patients from the high dependency unit in a tertiary referral university hospital were considered for inclusion if they had been admitted with moderate to severe pain following surgery or trauma, were aged between 18 and 75 years of age, and were receiving fentanyl by patient-controlled analgesia (PCA) for management of their acute pain. All patients underwent detailed workup including history, physical examination and laboratory investigations including haematology, coagulation, biochemistry, liver function and urine drug screen (DipScan, Point of Care Diagnostics Australia Pty Ltd, Artarmon, NSW). Reliable venous access in the left or right arm was required to allow collection of a number of blood samples.

Patients were given fentanyl by PCA because it is metabolised by the cytochrome P450 enzymes CYP3A4/5 and not CYP2D6, the latter being the main enzyme responsible for the metabolism of tramadol and production of O-desmethyltramadol (M1), the active metabolite of tramadol responsible for most of the drug’s opioid agonist effect. Informed consent was obtained from the patients the day before the study commenced.

### Exclusion criteria

Exclusion criteria for both the healthy subjects and patients included an allergy or hypersensitivity to opioids, current use of opioid or psychoactive medications (including sedatives, hypnotics or tranquillisers), current or previous alcohol or drug abuse, current or recent use of medications or herbal preparations known to induce or inhibit CYP2D6/CYP3A4 enzymes, renal impairment (defined as a calculated creatinine clearance of ≤ 75 ml/min), donation of blood within three months prior to the study, or any condition that would interfere with blood sampling or drug disposition [18].

Exclusion criteria also included abnormal liver function (in healthy subjects defined as outside the normal laboratory range; in the patient group defined as liver function tests > 3 times the upper limit of normal range). Healthy subjects were also excluded if the urine drug screen was positive for any opioids, benzodiazepines, amphetamines, cocaine or cannabis.

### Study design and procedures

#### Healthy cohort

A urine drug screen was performed on the morning before tramadol administration. An 18 gauge IV cannula (Introcan, Braun, Germany) was placed in a forearm or antecubital fossa vein for blood sampling. A 20 ml sample was withdrawn prior to tramadol (Tramal®, tramadol hydrochloride [CSL Limited]) administration for assay standards. Each subject received a single SC bolus dose of 50 mg of tramadol injected over 60 seconds through a 27 gauge needle sited just inferior to the clavicle on the contralateral side to the cannula. The SC needle was primed with tramadol prior to placement and the bolus dose administered immediately upon insertion.

Blood samples (5 ml) were collected from the venous cannula at 2, 5, 10, 15, 30, 60, 90, 120, 180, 240, 300, 360, 480, 600 minutes and 24 hours after tramadol dosing. Urine was collected from 0 to 10 hours after the dose was given so that tramadol, M1 and N-desmethyltramadol (M2) concentrations could be measured.

### Patients

The same protocol for tramadol administration and blood sampling was followed as for the healthy subjects. Acute Physiological and Chronic Health Evaluation (APACHE II) scores were calculated on the day of the study. The APACHE II score is a classification system designed to assess the severity of disease for adult patients admitted to an intensive care unit [19]. Urine was also collected cumulatively for the first 10 hours of the study for measurement of tramadol, M1 and M2 concentrations.

### Monitoring

During the study period, all patients and healthy subjects had respiratory and heart rates, sedation scores, temperature and the severity of nausea/vomiting recorded every 30 minutes for the first 3 hours, and then hourly for the duration of the study. Level of sedation was scored on a 4-point scale from 0 to 3 (0 = wide awake, 1 = rouses easily and can stay awake; 2 = rouses easily but has difficulty staying awake, and 3 = somnolent, difficult to rouse) [5]. The degree of nausea and vomiting/retching was scored on a scale from 0 to 3 (0 = none, 1 = mild and does not need treatment, 2 = treatment was effective, 3 = persists despite treatment). Peripheral oxygen saturation (SpO_2_) levels and respiratory rates were also monitored and the lowest values recorded. Any other adverse effects reported by the patients and healthy subjects were also documented.

### Processing of blood samples

The blood samples were stored in 10 ml heparinised plastic tubes and centrifuged within 2 hours of collection. Following centrifugation, the plasma was transferred into identically labeled polypropylene tubes. Samples were then frozen at -20°C and transferred to a -70°C freezer within 24 hours of processing. The samples were later transferred to the analytical laboratory and stored at -20°C for tramadol determination.

### Plasma and urine assays

Plasma and urine tramadol, M1 and M2 concentrations were determined by high-performance liquid chromatography (HPLC) with fluorescence detection and using an external standardisation procedure as no internal standard was readily available at that time. This then required exact volumes of all liquids as outlined below. Plasma sample preparation was as follows: plasma (500 μl) was alkalinised with 1 M (100 μl) sodium hydroxide prior to extraction in 3 ml hexane: ethyl acetate (80:20). The organic layer (2.7 ml) was then back extracted into 0.05 M (150 μl) hydrochloric acid, the organic phase was aspirated and 100 μl of the remaining acidic phase was injected on to the HPLC system. Urine sample preparation was as follows: samples were centrifuged (6 minutes, 14000 r.p.m.), the supernatant was diluted to 1 in 100 in mobile phase (details below) and injected (100 μl) on to the HPLC system. The retention time of tramadol was 5.4 minutes (plasma) and in urine was 8.0 minutes and M1 and M2 metabolites were 3.7 and 8.7 minutes, respectively following mobile phase modification. Peak areas were used as output responses.

The HPLC system was a LC Workstation Class LC10 (Shimadzu, Kyoto, Japan) consisting of a SIL-10ADvp auto injector and LC-10ADvp liquid chromatograph (pump), with fluorescence detection (excitation and emission wavelengths of 210 and 305 nm, respectively; LC-240 Perkin Elmer, Buckinghamshire, UK) and a C-R6A Chromatopac integrator (Shimadzu, Kyoto, Japan). Tramadol, M1 and M2 were separated on a C18 LUNA analytical column (150 × 4.6 mm, Phenomenex, Lane Cove, Australia). The mobile phase for urine samples consisted of acetonitrile: 25 mM di-potassium hydrogen phosphate (39:61, v/v) adjusted to pH 8.9 with 85% orthophosphoric acid at a flow rate of 1.0 ml/min, and the mobile phase for plasma samples consisted of acetonitrile: 25 mM di-potassium hydrogen phosphate (15:75, v/v) adjusted to pH 3.0 with 85% orthophosphoric acid at a flow rate of 1.5 ml/min.

Calibration curves for tramadol, M1 and M2 quantification from urine samples were constructed with 6 final concentrations (ng/ml) ranging from 500 to 5000, 10 to 475 and 75 to 475, respectively. Calibration samples were prepared identically in blank human urine. Low, medium and high QC samples of tramadol, M1 and M2 were also prepared with final concentrations (ng/ml) of 750, 75 and 75, respectively; 2500, 250 and 250, respectively; and 4750, 475 and 475, respectively. The inter- and intra-assay imprecision and inaccuracy of the assays (n = 6) based on the QC samples were all < 10%. At the LLOQ of 25 ng/ml, intraday imprecision was 8.7% and inaccuracy was 4.2%; at the low (75 ng/ml), medium (200 ng/ml) and high (350 ng/ml) QCs, the values were 6.5% and 1.6%, 9.0% and 2.7% and 6.7% and 4.3%, respectively (all n = 6). For interday imprecision and inaccuracy, the values were 17.4% and 5.8%, 6.9% and 2.9%, 9.2% and 6.4% and 2.6% and 2.8% (n = 6). There was no extraction required for the urine samples and the recovery was >95%.

**Figure 1:**
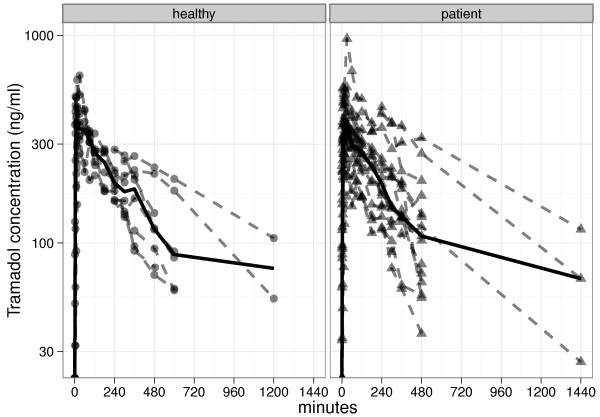
**Individual plasma tramadol concentrations in the healthy subjects and patients.** All patients and healthy subjects received a 50 mg subcutaneous bolus dose of tramadol. The median for each subject group is shown by the solid line. Graphs are truncated at 1200 minutes and 1440 minutes respectively, as plasma concentrations were less than the lower limit of quantification of the assay.

Calibration curves for tramadol quantification from plasma samples were constructed with 6 final concentrations ranging from 25 to 1000 ng/ml. Calibration samples were prepared identically in blank human plasma. Low, medium and high quality control (QC) samples of tramadol were also prepared with final concentrations (ng/ml) of 75, 200 and 350, respectively. Extraction recovery of tramadol was > 54.3%. The inter- and intra-assay (n = 6) imprecision and inaccuracy of the assays based on the QC analysis were all < 17.5%. The lower limit of quantification was 25 ng/ml. The protocols in both groups were exactly the same and blood samples were collected up to 24 hours after tramadol dosing. However, the curves were truncated because at certain time points, the concentrations fell below the limit of quantification (<LOQ) and were not easily detectable using the assay described (Figure 1).

The assay in both plasma and urine from patient samples revealed no additional peaks or shoulders on the chromatogram and chromatograms from other patient samples not administered tramadol contained no peaks at the retention times. Plasma and urine samples stored at -20°C for 3.6 and 9 months revealed no time-dependent changes in concentration.

### Non-compartmental analysis of pharmacokinetics

The data were analysed using a non-compartmental approach (NCA) based on analysis of calculated and extrapolated area under the curve (AUC_0-∞_) [20]. The AUC from zero time to the last quantifiable concentration was calculated by trapezoidal integration (linear up, log down method) and extrapolated to time infinity by assuming an exponential decline (best fit of 3 to 6 data points) to estimate the terminal exponential rate constant and therefore terminal half-life (t½). The analysis used previously validated scripts written in the R language [21]. Other pharmacokinetic parameters including time taken to reach maximum plasma concentrations (t_max_), maximum plasma concentration (C_max_), t½ and mean residence time (MRT) were also calculated from the observed data. Urine concentrations of tramadol, M1 and M2 metabolites and total urine volume were used to calculate the total amount of each excreted in 10 hours.

### Statistical analysis

Demographic data are expressed as median (range) and were analysed using a two-tailed unpaired t-test. Pharmacokinetic data analysis used the R data analysis and statistical language (Version 2.10.1) using a single factor ANOVA [21]. Values were expressed as mean (±SD), or as mean (range) depending on their distribution.

## Results

The study was completed safely with no tramadol-related adverse effects reported in either group. Of the 10 healthy subjects recruited, two did not attend on the designated study day and the two reserves were also unavailable, hence eight were studied. Two of the patients were given a single dose of oxycodone during the study period. Their data were not excluded from analysis as oxycodone does not inhibit CYP2D6 (see discussion).

The demographics of the two study groups are summarised in Table 1. All the eight subjects in the healthy cohort were male: median age 25 years (range 19 to 51), median weight 74 kg (range 60 to 97). The median age of the 15 patients (13 male and two female) was 43 years (range 20 to 65), median weight was 75 kg (range 72 to 76) and median Acute Physiological and Chronic Health Evaluation II score was 7 (range 2 to 14). The healthy group was significantly younger than the patients (p = 0.006). There was no difference in weight between the two groups. Diagnoses included 10 patients with multi-trauma, one patient who underwent orthopedic surgery and four patients who had gastrointestinal surgery.

**Table 1:** Demographic and clinical details of patients and healthy subjects

**Demographic and clinical details**		**Patients n = 15**	**Healthy subjects n = 8**
**Age (years)**		43 (20–65)*	25 (19–51)
**Gender**	**Male**	13	8
	**Female**	2	0
**Weight (kg)**		75 (72–76)	74 (60–97)
**APACHE II score†**		7 (2–14)	Not applicable
**Admission Diagnosis (n)**		Multi-trauma (10)	Not applicable
		Orthopedic surgery (1)	
		Gastrointestinal surgery (4)	

All patients and healthy subjects received 50 mg subcutaneous bolus dose of tramadol. Data are shown as median (range) *p = 0.006 † Acute Physiological and Chronic Health Evaluation.

No abnormalities were detected in any investigations performed at either the pre-study or post-study screening. None of the healthy subjects or patients showed clinically significant changes in blood pressure, SpO_2_ levels, heart rate, respiratory rate or body temperature during the study period, and none became sedated or had any nausea or vomiting.

### Pharmacokinetics

**Figure 2:**
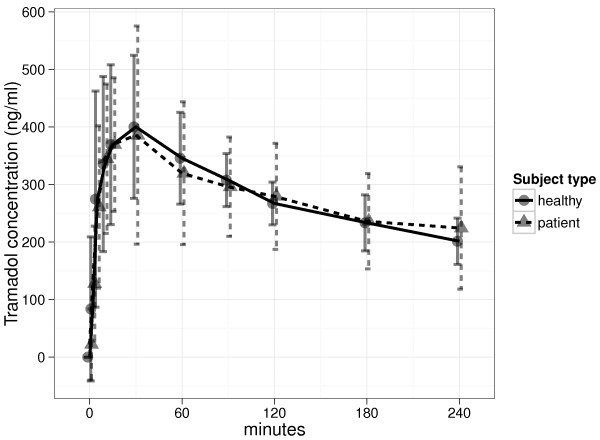
**Mean plasma concentration times in healthy subjects (****) and patients (****).** All patients and healthy subjects received a 50 mg subcutaneous bolus dose of tramadol. Data are shown as mean and standard deviation. The time scale is reduced compared to Figure 1 to emphasise the absorption phase.

The tramadol blood concentration-time curves for the individuals in each group are summarised in Figure 1 and the mean plasma concentration times are shown in Figure 2. There were no significant differences in AUC_0-∞_ between the healthy subjects and the patients. The non-compartmental pharmacokinetic parameters are summarised in Table 2. In both cohorts, absorption of tramadol after SC administration was rapid. There were no significant differences in any of the pharmacokinetic variables (AUC_0-∞_, p = 0.96; t_max_, p = 0.73; C_max_, p = 0.67; t½, p = 0.96; MRT, p = 0.97). The range of t_max_ values was 5–60 minutes in both the healthy subjects and the patients.

**Table 2:** Pharmacokinetic parameters

**Parameter/ units**	**Healthy subjects mean (SD), (n = 8)**	**Patients mean (SD), (n = 15)**	**p value**	**Differences between patients and healthy subjects**	
				**Mean**	**Standard error**
**C**_**max**_**(mcg/ml)**	0.47 (0.13)	0.44 (0.18)	0.67	-0.032	0.073
**AUC**_**∞**_(mcg/ml*min)	175 (75)	177 (109)	0.96	2.07	43.6
**t½ (min)**	310 (147)	306 (164)	0.96	-3.5	69.6
**t**_**max**_**(min)**	20.6 (18.8)	23.3 (17.1)	0.73	2.7	7.7
**MRT (min)**	466 (224)	463 (233)	0.97	-3.4	100.8

All eight healthy subjects and 15 patients received 50 mg tramadol as a subcutaneous bolus injection. The p-value is for a One Factor ANOVA comparing the cohorts. The mean difference between the patient and healthy groups is shown with its standard error as an indicator of study power.

*represents the unit of AUC as in product of ml and min.

Urinary excretion of tramadol, M1 and M2 metabolites in both cohorts in our study were measured. They showed respective mean values of 28.2% tramadol, 4.01% M1 and 1.01% M2 in the urine collected over 10 hours in the healthy subjects. In the patient cohort, values were 24.3% tramadol, 3.43% M1 and 2.02% M2. The mean M1/tramadol excreted dose ratios were 0.14 ± 0.12 (range 0.01 to 0.34) for healthy subjects and 0.17 ± 0.14 (range 0.02 to 0.51) for the patient cohort. The mean M2/tramadol excreted dose ratios were 0.04 ± 0.03 (range 0.01 to 0.07) for healthy subjects and 0.08 ± 0.09 (range 0.06 to 0.33) for the patient cohort.

## Discussion

This study describes the pharmacokinetics of a single subcutaneous dose of tramadol and shows that there were no differences in the rates of absorption or drug exposure between healthy subjects and patients. Although the pharmacokinetic profile of tramadol has previously been characterised in humans after oral, IM and IV administration, similar data have not been available for the SC route [6-9]. While these data are frequently determined in healthy subjects they cannot always be extrapolated to sick patients, hence the importance of also documenting pharmacokinetics in this patient cohort [22].

Tramadol is an atypical centrally acting analgesic agent which acts as both a mu-opioid receptor agonist and inhibitor of noradrenaline and serotonin reuptake. It is a racemic mixture where the (+) enantiomer preferentially inhibits serotonin reuptake and the (-) enantiomer preferentially inhibits noradrenaline reuptake [23]. Tramadol is metabolised by O-demethylation to the active M1 and N-demethylation to the inactive M2 metabolites. Metabolism is dependent on the cytochrome P450 enzymes CYP2D6 and CYP3A4 [23,24]. M1 displays a 200-fold higher affinity for the mu-opioid receptor than the parent drug [25]. Tramadol and its metabolites are almost completely excreted by the kidney [23].

Tramadol is effective in the management of acute pain, although it may be inadequate for the management of moderate to severe acute pain if used as the sole analgesic agent [1,10]. When combined with morphine, tramadol is opioid-sparing, but the effect is infra-additive [26]. Since tramadol is less likely to lead to respiratory depression and reduced gastrointestinal motility compared with pure opioid agonists at equianalgesic doses, it may be a useful choice in some patients such as those with respiratory compromise or obstructive sleep apnea, or those who have had gastrointestinal surgery [1,23,27,28]. Its effectiveness in the treatment of acute neuropathic pain may also make it a worthwhile agent in some patients [29,30].

### Pharmacokinetics

While subcutaneous tramadol would not be of interest in mechanically ventilated critically ill patients where intravenous sedation and analgesia is a requirement, subcutaneous pain relief can be useful in spontaneously ventilating patients in a high dependency setting [3,4]. It may avoid the need for IV access and reduce the incidence of respiratory and gastrointestinal side effects when compared to IV administration [2]. However, the pharmacokinetics of drugs cannot be presumed to be the same in severely ill patients as in healthy subjects. Trauma, the perioperative state and critical illness may affect drug absorption and distribution due to differences in skin perfusion and cardiac output. In a similar study investigating the pharmacokinetics of oxycodone after a single SC dose, marked differences were revealed between healthy subjects and a similar cohort of patients [22]. The latter showed that AUC_0-∞_, C_max_ and t½ were all significantly lower in the patient cohort despite no differences in t_max_ or MRT. In contrast, in this study of SC tramadol, no significant differences were found in AUC_0-∞_ t½, t_max_, C_max_ or MRT between patients and healthy subjects.

The SC pharmacokinetics of absorption of the following opioids has previously been reported: morphine in healthy subjects and older patients; fentanyl in healthy subjects; and oxycodone in both healthy subjects and critically ill patients [22,31-33]. The mean t_max_ values for tramadol in this study of 20.6 and 23.3 minutes in healthy subjects and patients, respectively, were comparable to those reported for oxycodone in both healthy subjects and critically ill patients (22.1 and 20.5 minutes respectively), but longer than that reported for fentanyl and morphine in subjects (both 15 minutes) or morphine in elderly patients (15.9 minutes).

In comparing the pharmacokinetic parameters of tramadol after oral, SC, IV and IM routes of administration in healthy subjects, our study using the SC route of 50 mg tramadol showed that the t_max_ was 20.6 minutes, t½ was 5.2 hours, C_max_ was 0.47 mcg/ml and AUC was 175 mcg/ml*min. Previous reports have shown that a 100 mg oral dose of tramadol in healthy subjects gave a t_max_ value of 66 minutes, a t½ value of 5.6 hours, a C_max_ value of 0.31 mcg/ml and an AUC value of 160 mcg/ml*min, whilst a single IV dose of 50 mg tramadol gave a t½ value of 5.5 hours, a C_max_ value of 0.35 mcg/ml and an AUC of 93 mcg/ml*min. With a single IM dose of 50 mg tramadol, the t_max_ was 45 minutes, t½ was 5.5 hours, C_max_ was 0.19 mcg/ml and the AUC was 95 mcg/ml*min [9].

There are limited data on the urinary excretion of tramadol and its metabolites. In healthy subjects given a 50 mg oral dose of tramadol, the mean values for urinary excretion over 24 hours (expressed as a percentage of the dose administered) were tramadol 12%, M1 15%, and M2 4% [34]. Following a 100 mg dose of oral tramadol, also given orally to healthy subjects, with urine collected over 30 hours, the mean values were 16.2% unchanged tramadol, 11.2% M1, and 1.1% M2 [35]. Similar results were reported by Rudaz et al. also following administration of 100 mg oral tramadol to healthy subjects, again with urine collected over 30 hours: 16% unchanged tramadol, 16% M1 and 2.2% M2 [36]. In our study, respective mean values were 28.2% tramadol, 4.01% M1 and 1.01% M2 in the urine collected over 10 hours in the healthy group. In the patient cohort, values were 24.3% tramadol, 3.43% M1 and 2.02% M2. Since the clearance via the M1 and M2 pathways does not appear to be different between the two groups (as assessed by the urinary metabolite to tramadol ratio), this suggests that the CYP2D6 (M1) and CYP3A4 (M2) pathways were not altered in severely ill patients.

The study was designed to avoid pharmacokinetic interactions with other drugs. In the patients fentanyl was chosen as the “rescue” opioid, delivered by PCA, for intercurrent pain relief because it is metabolised by the cytochrome P450 enzyme CYP3A4. CYP3A4 is also responsible for metabolism of tramadol to M2. However, fentanyl is only a substrate and not an inducer/inhibitor of CYP3A4 at therapeutic concentrations. If fentanyl was competing with tramadol as a substrate for this enzyme, the AUC_0-∞_ results for tramadol would have been higher in the patients who were receiving fentanyl. Two of the patients received oral oxycodone during the study period. Oxycodone is metabolised by CYP3A4 and CYP2D6. Similarly, oxycodone is only a substrate and not known to be an inhibitor of CYP2D6, and therefore the results from these patients were not excluded.

A possible limitation of this study is the mismatching between the group in terms of age and sex. However given that no difference was noted in the pharmacokinetics between the groups it is unlikely that the results of the study would have been different if the groups had been better matched. Based on the paucity of pharmacokinetic data after SC administration of this drug, no a priori power calculation was performed, which is therefore one of the limitations of this study. Hence, between-group differences in some pharmacokinetic parameters, found to be similar in this study, cannot be confidently excluded as the study may not have been adequately powered to demonstrate a difference. Nevertheless, there is no trend for a difference in any parameter so any possible difference is likely to be small and may not be clinically relevant. The estimated effect size and its standard error are given in Table 2 to indicate the study power. Moreover, there may be some patient groups where the time-course of drug concentrations and effect are clinically different from the patients in the current study. We chose not to phenotype our subjects for CYP2D6, because total subject numbers would have been too small to detect significant differences.

## Conclusions

The rate of absorption, time to peak blood concentration, and the rate of elimination of tramadol following SC injection were similar in healthy subjects and severely ill patients. The time to peak absorption was also similar to pure opioid agonists (morphine, fentanyl and oxycodone) given by SC injection. As the key pharmacokinetic parameters governing overall tramadol exposure (AUC_0-∞_) and the peak concentrations (C_max_, t_max_) did not differ, these data show that the same dosing regimens (dose and frequency of administration) for SC tramadol administration may be used in both healthy subjects and in severely ill patients.

## Abbreviations

IV: Intravenous; IM: Intramuscular; SC: Subcutaneous; BMI: Body mass index; PCA: Patient controlled analgesia; M1: 0-desmethyltramadol; M2: N-desmethyltramadol; CYP: Cytochrome P 450 enzymes; APACHE: Acute physiological and chronic health evaluation; HPLC: High performance liquid chromatography; QC: Quality control; LOQ: Limit of quantification; NCA: Non compartmental approach; AUC: Area under the curve; t½: Terminal half-life; tmax: Time taken to reach maximum plasma concentration; Cmax: Maximum plasma concentration; MRT: Mean residence time.

## Competing interests

No external funding and no competing interests declared.

## Authors’ contributions

ND conducted study in healthy volunteers, assisted in data collection and manuscript drafting. KS conducted study in patients, assisted in data collection and manuscript drafting. TR conducted study in patients, assisted in data collection, manuscript drafting and manuscript submission. AS conducted pharmacokinetic analysis, assisted in data analysis, interpretation and critical revision of manuscript. RU designed the study, involved in pharmacokinetic analysis, interpretation of data, assisted in manuscript drafting. SC assisted in conducting study in patients, data collection and manuscript drafting. JO contributed to experimental design and plan, assisted in conducting study in healthy volunteers and patients, data collection and manuscript drafting. MC designed the study in patients, assisted in conducting the study and manuscript drafting. GL designed the study in healthy volunteers, assisted in data interpretation and manuscript drafting. All authors read and approved the final manuscript.

## Pre-publication history

The pre-publication history for this paper can be accessed here:

http://www.biomedcentral.com/1471-2253/14/33/prepub
